# Ketogenic diet in pharmacoresistant epilepsies: a clinical nutritional assessment

**DOI:** 10.1055/s-0044-1779269

**Published:** 2024-02-23

**Authors:** Cecilia Nascimento de Mendonça, Adelia Maria de Miranda Henriques-Souza, Larissa de Andrade Viana, Paula Azoubel de Souza, Luis Bandeira Alves Neto, Maria Júlia Gonçalves de Mello

**Affiliations:** 1 Instituto de Medicina Integral Prof. Fernando Figueira, Programa de Graduação Stricto Sensu do IMIP, Recife PE, Brazil.; 2 Instituto de Medicina Integral Prof. Fernando Figueira, Centro de Terapias Cetogênicas do IMIP, Recife PE, Brazil.

**Keywords:** Seizures, Diet, Ketogenic, Malnutrition, Drug Resistant Epilepsy, Convulsões, Dieta Cetogênica, Desnutrição, Epilepsia Resistente a Medicamentos

## Abstract

**Background** Epilepsies are among the most prevalent chronic neurological diseases, usually beginning in childhood. About 30% of children with epilepsies develop seizures that are difficult to control with medication. Recurrent epileptic seizures hinder diet intake, impairing the nutritional status. Although non-pharmacological interventions (e.g., ketogenic diet therapy) can improve epileptic seizure frequency, few studies analyzed their impact on the nutritional status of children and adolescents with epilepsies.

**Objective** The aim was to evaluate the effects of a ketogenic diet on the nutritional status and clinical course of patients with pharmacoresistant epilepsies.

**Methods** This cross-sectional study included patients under 18 years of age followed up at the Ketogenic Diet Ambulatory Clinic of the Instituto de Medicina Integral Prof. Fernando Figueira between December 2015 and December 2021. Socioeconomic, clinical, nutritional, and laboratory data were collected from medical records at different time points during the ketogenic diet.

**Results** The sample comprised 49 patients aged between 5 months and 17 years (median = 4.4 years), mostly male (62.1%), and from Recife and the metropolitan region (51%). Underweight patients (BMI-for-age) improved their nutritional status in six months. However, patients who were normal weight and overweight maintained their nutritional status. Dyslipidemia was a common and short-term adverse effect. Moreover, the treatment decreased epileptic seizure frequency and antiseizure medication intake.

**Conclusion** The ketogenic diet prevented malnutrition from worsening and reduced epileptic seizures and antiseizure medication intake.

## Resumo

**Antecedentes** A epilepsia, uma das doenças neurológicas crônicas mais prevalentes, tem geralmente início na infância. Cerca de 30% das crianças com epilepsia desenvolvem crises de difícil controle medicamentoso. As crises epilépticas recorrentes dificultam a ingestão alimentar, prejudicando o estado nutricional. Intervenções não farmacológicas, como a terapia com dieta cetogênica, podem melhorar a frequência das crises epilépticas, mas existem poucos estudos sobre a repercussão no estado nutricional da criança/adolescente.

**Objetivo** Avaliar o efeito da terapia cetogênica sobre o estado nutricional e a evolução clínica da epilepsia fármaco-resistente.

**Métodos** Estudo tipo corte transversal envolvendo menores de 18 anos acompanhados no Ambulatório de Dieta Cetogênica do Instituto de Medicina Integral Prof. Fernando Figueira entre dezembro de 2015 e dezembro de 2021. Dados socioeconômicos, clínicos, nutricionais e laboratoriais foram coletados nos prontuários dos pacientes em vários momentos da terapia cetogênica.

**Resultados** A amostra foi composta por 49 pacientes com idades entre cinco meses e 17 anos (mediana = 4,4 anos), a maioria do sexo masculino (62,1%) e procedentes de Recife e região metropolitana (51%). Pacientes com baixo peso (de acordo com o IMC para idade) melhoraram seu estado nutricional em seis meses. No entanto, os pacientes com peso adequado e com sobrepeso mantiveram seu estado nutricional. A dislipidemia foi um efeito adverso frequente e de curta duração. Além disso, o tratamento reduziu a frequência de crises epilépticas e a dose de fármacos anticrises.

**Conclusão** A dieta cetogênica preveniu o agravamento da desnutrição e reduziu as crises epilépticas e a dosagem de fármacos anticrises.

## INTRODUCTION

Epilepsies are one of the most common neurological diseases. 1 Epilepsies can be classified as pharmacoresistant when two antiseizure medications (ASMs), taken as prescribed and properly chosen, whether monotherapy or combined, fail to stop or control the seizures. 2 Thus, patients with pharmacoresistant epilepsies benefit from non-pharmacological or non-conventional interventions, such as the ketogenic diet (KD). 3

Severe epileptic syndromes impair nutritional status. However, the relationship between malnutrition and epilepsies is yet to be determined. 4 Studies suggest muscle spasms increase energy expenditure, and ASMs hamper nutrient absorption. 5 In this sense, KD helps the nutritional treatment of patients with pharmacoresistant epilepsies using a high-fat, low-carbohydrate, and adequate-protein approach in a 3:1 or 4:1 ratio (3 or 4 grams of fat to 1 gram of protein + carbohydrate). 6 Other less restrictive and palatable KD may also be used, such as modified Atkins (MAD) and low-glycemic-index diets (LGI). 7 8

KD aims at improving the clinical course of epilepsies and reducing ASMs dosage and adverse effects. Also, this diet reduces treatment costs, improves the quality of life, promotes the well-being of families, and reduces the risk of sudden death. 3 Although KD reduces epileptic seizures, its effects on the nutritional status of pediatric and adolescent patients need verification. 9 Therefore, this study aimed to evaluate the effects of KD on the nutritional status and clinical course of patients with pharmacoresistant epilepsies.

## METHODS

This cross-sectional retrospective study included patients under 18 years diagnosed with pharmacoresistant epilepsies. Patients were followed up for at least six months at the Ketogenic Diet Ambulatory Clinic of the Instituto de Medicina Integral Prof. Fernando Figueira (IMIP) from December 2015 to December 2021. Patients who started KD in other services were excluded from the sample, and those with incomplete medical records were considered losses.

The IMIP is in Recife, the capital of the state of Pernambuco, and is one of the most important hospital structures in Northeast Brazil (the region with the lowest socioeconomic development). The Ketogenic Diet Ambulatory Clinic for Pharmacoresistant Epilepsies was established in December 2015, is the first in North and Northeast Brazil, and has trained professionals in Child Neurology and Nutrition.

Caregivers were informed about the study via telephone. If they chose to participate, their consent was recorded and signed by a witness that was not from the research team and who accompanied the interview. The researcher kept this document and delivered it to the caregiver on their next visit to the clinic or sent a copy via WhatsApp or telephone message (SMS).

Socioeconomic, clinical, nutritional, and laboratory data were collected from medical records. The socioeconomic variables involved the age, sex, and origin of the patients. Clinical variables comprised epilepsies etiology according to the International League Against Epilepsy – ILAE criteria, 10 number of daily epileptic seizures, ASMs use and dosage, and ketosis measurement. Ketosis state was assessed using the daily measure of ketonuria, indicated by +++ urine ketones or levels above 150 mg/dL. 11 12

At each consultation, a team of nutritionists assessed the nutritional status; results were analyzed in the follow-up periods. Nutritional status was assessed using body mass index (BMI)-for-age. 13 14 Total serum cholesterol ≥ 170 mg/dL characterized hypercholesterolemia, while triglycerides > 100 mg/dL for patients up to ten years and > 130 mg/dL for those over ten years characterized hypertriglyceridemia. 15 16

Data on KD characteristics (KD ratio [transition, 2:1; 3:1; 4:1, MAD, or LGI], feeding route [oral or enteral via nasogastric tube or gastrostomy], duration, treatment status, and adverse effects) were collected at different time points (3, 6, 12, 18, and 24 months of KD). Treatment was complete when patients achieved well-controlled epilepsies and discontinued when patients presented adverse effects or no improvement in epileptic seizures. Also, the treatment was discontinued or abandoned if the family did not attend the consultations.

Epileptic seizure frequency was reported by caregivers using a calendar. These data were compared to baselines obtained previously (frequency per month before KD). Moreover, response to KD was measured after three, six, 12, 18, and 24 months. Reduction in epileptic seizure frequency was classified according to the Huttenlocher criteria 17 ; a reduction of over 50% indicated well-controlled epilepsies. 18 19

Descriptive analysis was performed using Stata ^®^ software, version 13.0. The relative frequencies were used for categorical variables, and central tendency and dispersion measures for continuous variables.

The study followed Resolution No. 466/2012 and Circular Letter No. 2/2021/CONEP/SECNS/MS (Brazil) concerning research in digital media and was approved by the research ethics committee of the Instituto de Medicina Integral Professor Fernando Figueira (no.: 50599821.0.0000.5201).

## RESULTS

The eligibility criteria were applied to 81 patients. Five patients were excluded because they started KD in another service, and 27 were lost due to unavailable contact or lack of information on medical records. Therefore, the study included 49 patients (men = 62.1%) between five months and 17 years of age (median of 4.4 years).

Most patients were from Recife (state capital) and metropolitan region (51.0%), followed by the inland of Pernambuco (38.8%) and other states of Northeast Brazil (10.2%). According to medical records, the pharmacoresistant epilepsies etiology was likely genetic (55.1%), structural (26.5%), infectious (12.2%), or unknown (6.1%).

The feeding route, KD duration, and treatment status at the end of the study are shown in Table 1 . About 77.6% of the patients presented adverse effects. Most occurred in the short term (less than three months) and were related to the gastrointestinal tract (nausea, vomiting, constipation, diarrhea, and lack of appetite); sleepiness and irritability occurred in low proportion. These data are not presented in the table.

**Table 1:** Characterization of the ketogenic diet performed by 49 patients with pharmacoresistant epilepsies followed at IMIP

		n (%)
Feeding route	Oral	39 (79.6)
	Enteral via nasogastric tube	2 (4.1)
	Enteral via gastrostomy	8 (16.3)
**Diet duration (months)**	3 to 6	6 (12.2)
	7 to 12	15 (30.6)
	13 to 24	11 (22.5)
	25 to 36	5 (10.2)
	Over 36	12 (24.5)
**Treatment status in December 2021**	Followed up	21 (42.9)
	Completed	8 (16.3)
	Discontinued*	7 (14.3)
	Discontinued by the family**	13 (26.5)
**Adverse effects**	Yes	38 (77.6)
	No	11 (22.4)

Notes: *Discontinued due to adverse effects or lack of improvement;**Treatment discontinued by family choice.

**Figure 1:**
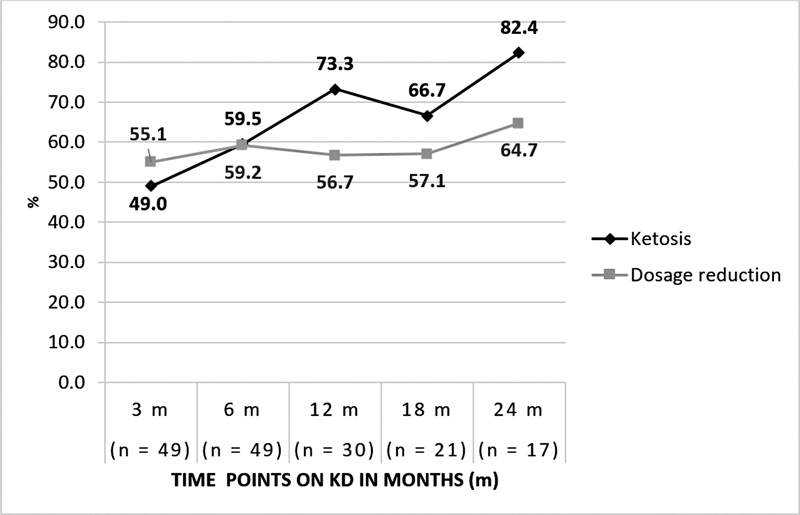
Percentage of ketosis (urinary ketones > 150mg/dL) and reduction in the antiseizure medication dosage.

Table 2 shows the KD ratio modified at each time point according to the clinical response and the estimated percentage of reduced epileptic seizure frequency. The number of patients gradually reduced in each period. At six months of treatment, 69.3% of the patients showed a reduction in epileptic seizure frequency by over 50%, 14.3% by below 50%, and 16.3% showed no reduction. Seventeen patients were under KD for 24 months, and two (11.8%) had poorly controlled epilepsies. Figure 1 shows the ketosis and ASMs dosage reduction at each time point.

**Table 2:** Ketogenic diet ratio and reduced epileptic seizure frequency according to time points on KD.

		Time points on KD (months)				
		3 n = 49	6 n = 49	12 n = 30	18 n = 21	24 n = 17
		n (%)	n (%)	n (%)	n (%)	n (%)
Type of KD	Transition or 1:1	2 (4.1)	3 (6.1)	−	2 (9.5)	1 (5.9)
	2:1 or 2.5:1	16 (32.7)	9 (18.4)	4 (13.3)	1 (4.8)	−
	3:1 or 3.5:1	14 (28.6)	14 (28.6)	6 (20.0)	2 (9.5)	3 (17.7)
	4:1 or 4.5:1	13 (26.5)	18 (36.7)	16 (53.3)	13 (61.9)	12 (70.6)
	MAD	4 (8.2)	3 (6.1)	3 (10.0)	3 (14.3)	1 (5.9)
	LGI	−	2 (4.1)	1 (3.3)	−	−
Classification of the reduction in epileptic seizure frequency (per month)	Excellent (100%)	8 (16.3)	10 (20.4)	5 (16.7)	3 (14.3)	4 (23.5)
	Very good (90% - 99%)	7 (14.3)	11 (22.4)	5 (16.7)	3 (14.3)	7 (41.2)
	Good (50% - 90%)	16 (32.7)	13 (26.5)	9 (30.0)	6 (28.6)	4 (23.5)
	Regular (< 50%)	11 (22.5)	7 (14.3)	8 (26.7)	5 (23.8)	1 (5.9)
	No reduction	7 (14.3)	8 (16.3)	3 (10.0)	4 (19.1)	1 (5.9)

Abbreviations: LGI, low-glycemic index diet; MAD, modified Atkins diet.

**Figure 2:**
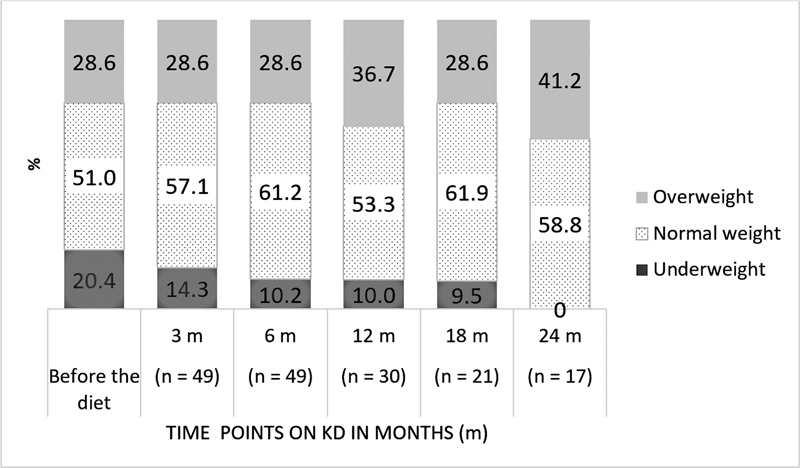
Nutritional status (BMI-for-age) according to time points on KD of patients with pharmacoresistant epilepsies.

Nutritional status (BMI-for-age) is presented in Figure 2 . Patients before the KD presented normal weight (51.0%), overweight (28.6%), and underweight (20.4%). Five of ten patients who were underweight presented normal weight after six months of KD; no patient presented overweight after the same period. Among the 17 patients who remained on the KD for 24 months, 58.8% were normal weight, and 41.2% were overweight.

Table 3 shows the evolution of the lipid profile. After 12 months of KD, the number of patients with hypertriglyceridemia and hypercholesterolemia increased, decreasing at 18 and 24 months.

**Table 3:** Lipid profile according to time points on KD.

Before the diet n = 4			3months n = 49	6months n = 49	12months n = 30	18months n = 21	24months n = 17
		n (%)	n (%)	n (%)	n (%)	n (%)	n (%)
Total cholesterol	Adequate	25 (51.0)	14 (28.6)	11 (22.5)	9 (30.0)	8 (28.1)	7 (41.2)
	Inadequate*	21 (42.9)	20 (40.8)	25 (51.0)	15 (63.3)	10 (47.6)	9 (52.9)
	No registry	3 (6.1)	15 (30.6)	13 (26.5)	2 (6.7)	3 (14.3)	1 (5.9)
Triglycerides	Adequate	31 (63.3)	20 (40.8)	20 (40.8)	16 (53.3)	11 (52.4)	7 (41.2)
	Inadequate**	15 (30.6)	13 (26.5)	16 (32.7)	12 (40.0)	7 (33.3)	8 (47.0)
	No registry	3 (6.1)	16 (32.7)	13 (26.5)	2 (6.7)	3 (14.3)	2 (11.8)

Abbreviation: IMIP, Instituto de Medicina Integral Prof. Fernando Figueira. Notes: * Hypercholesterolemia (≥ 170 mg/dL) 15 16 ; **Hypertriglyceridemia (> 100 mg/dL for patients up to 10 years and > 130 mg/dL for those over 10 years).

## DISCUSSION

In this study, KD prevented malnutrition worsening and reduced epileptic seizure frequency and ASMs dosage. Most patients with epilepsies were men, corroborating the literature. 20 21 Despite physical and neurological limitations, most patients performed oral feeding, a more physiological feeding route. However, in oral feeding patients, a low-palatability KD may cause treatment refusal and interruption. 7

All KD was outpatient and started with fat offered in low proportions and gradual progression, favoring palatability and adherence. Also, the diet became more restrictive as the fat proportion increased. After three months, about half of the patients presented ketosis (urinary ketones > 150 mg/dL), a frequency that increased according to KD duration and adjustments to optimize the ketogenic effect. High-fat diets (3:1 or 4:1 ratio) promoted the highest levels of ketosis.

A high prevalence of patients on the 4:1 ratio diet was observed after 24 months. A randomized clinical trial with 158 children on KD (4:1 ratio), modified Atkins diet, and the low-glycemic index diet showed that all diets reduced epileptic seizure frequency. 22

About 40% of the patients discontinued the KD: 14.3% due to adverse effects or lack of improvement in epileptic seizure frequency, and about 25% did not attend consultations. In a review of the applicability of KD, the dropout rate was between 20% and 54%. 22 This occurred because families had difficulties preparing and administrating the KD and attending consultations. 22 In our study, many patients were from the inland of Pernambuco or other states (covering distances > 500 kilometers) and did not receive financial support to access the reference center, corroborating the literature. Studies also reported discontinuation because of delayed improvement of epilepsies, adverse effects, and non-adherence due to low palatability. 7 22 23

Most patients (75%) presented gastrointestinal adverse effects (nausea, vomiting, diarrhea, and constipation), possibly due to intolerance to KD. Sleepiness and irritability were also presented in low frequency in the first months of treatment. Families were instructed on these mild adverse effects after KD adjustments. 7

A systematic review included 11 studies (n = 778 participants) on the effects of KD on pharmacoresistant epilepsies. The main reasons for discontinuation were adverse effects and non-adherence. All studies reported adverse effects, mainly short-term gastrointestinal symptoms. 24 Other less common effects were anorexia, lethargy, lower respiratory tract infections, and hyperammonemic encephalopathy. 24

In a randomized clinical trial involving 48 children with epilepsies (26 in KD and 22 on ASMs, about 58% of the KD group completed the 16-month follow-up. Despite the high cost of treatment, the amount and severity of epileptic seizure frequency were reduced. However, data were inconclusive regarding the best cost-benefit among KD and ASMs. 25

KD is used for rapid weight loss 26 ; however, weight loss is not the aim for pharmacoresistant epilepsies. Patients who were underweight, overweight, and with obesity may benefit from an ideal caloric value, according to age-adjusted protein-calorie needs, favoring the improvement or maintenance of their nutritional status. 7

In this study, half of the patients who were underweight before KD increased their BMI-for-age at six months of treatment, being classified as normal weight. Moreover, the percentage of patients who were overweight was maintained. The reduction in the sample may explain changes in the nutritional status after six months of KD. Most patients in each duration point had normal weight.

The effects of KD on nutritional status are still controversial. A study showed that weight gain in children with epilepsies on KD is similar to healthy children. 27 However, early and long-term KD may highly impair anthropometric values and bone mass, highlighting nutritional status monitoring concerns. 28 29 30 31

The lipid profile complements the nutritional status assessment. 32 Despite the prolonged and high fat intake, this study indicated that the lipid profile alternated between adequate and inadequate after 12 months of KD. The human body presents compensatory mechanisms to maintain balance in fat metabolism. 33 Less saturated fat intake may favor a normal lipid profile and controlled atherogenic fractions. 34 In addition, dyslipidemia is a common and short-term adverse effect of KD, occurring at any time and controlled with adequate monitoring and adjustment. 7 Statins may also be used if dyslipidemia remains severe and persistent. 7

KD is an unconventional, safe, and effective treatment that reduces epileptic seizures and the use of ASMs. 35 36 37 38 A multicenter study indicated that 33% of children with pharmacoresistant epilepsies achieved complete control, and 33% decreased ASMs use. In addition, a longitudinal study demonstrated reduced ASMs use; about 15% of the children could live without these epileptic seizures. 36 37

Most patients in our study showed reduced epileptic seizure frequency by over 50% after six months of treatment, corroborating the literature. In a multicenter study, 54% of the 51 children who presented a mean of 230 epileptic seizures per month showed a decrease by 50% after KD within three months of follow-up. 38

KD is efficient if epileptic seizure frequency is reduced by at least 50%. 18 19 Although some patients did not reach this percentage, reducing epileptic seizure frequency improved the cognitive and neurodevelopment, behavior, and quality of life of the patient and family, justifying the maintenance of treatment. 7

Although no difference was found in the mean number of ASMs used before and after KD, 6% of our patients showed dosage reduction, and one patient (2%) stopped using ASMs. In another study, the dosage reduction occurred in 33% of the sample. 39 Drug polytherapy is high-cost and presents adverse effects, limiting its use. 36 Moreover, a high dosage of ASMs impacts the behavior and cognition of children, justifying early withdrawal or reduction. 36 A study evaluated the main adverse effects of drug polytherapy using the perception of parents, indicating altered behavior, increased irritability, depressive symptoms, changes in cognitive function, motor and coordination problems, visual changes, headache, and dermatological, gastrointestinal, and hormonal complaints. 19

This study presents some limitations. The sample was small, and many losses occurred due to the lack of information in medical records. In addition, losses occurred because some patients were still undergoing treatment or did not adhere to KD and gave up before 24 months.

KD requires dedication, work, and organization, and most adverse effects can be prevented and controlled. 7 Important steps must be verified for treatment success: the initial lipid profile; the record of epileptic seizures; replacing drugs containing sugar; the type of diet to be followed; and products that are not part of the eating habit. In the follow-up, ketosis control is essential during the month before the consultation. Therefore, the family must participate in the treatment for the patient to feel included in the family context. 5 7

In conclusion, KD prevented malnutrition worsening and reduced epileptic seizures and ASMs use. Changes in the lipid profile occurred, and adverse effects were reported; however, they were short-term and controlled with diet adjustments.

## References

[1] Fisher R S Acevedo C Arzimanoglou A ILAE official report: a practical clinical definition of epilepsy Epilepsia 2014 55 04 475 482 24730690 10.1111/epi.12550

[2] Kwan P Arzimanoglou A Berg A T Definition of drug resistant epilepsy: consensus proposal by the ad hoc Task Force of the ILAE Commission on Therapeutic Strategies Epilepsia 2010 51 06 1069 1077 10.1111/j.1528-1167.2009.02397.x 19889013

[3] Charlie Foundation, Practice Committee of the Child Neurology Society Practice Committee of the Child Neurology Society International Ketogenic Diet Study Group Kossoff E H Zupec-Kania B A Amark P E Optimal clinical management of children receiving the ketogenic diet: recommendations of the International Ketogenic Diet Study Group Epilepsia 2009 50 02 304 317 18823325 10.1111/j.1528-1167.2008.01765.x

[4] Galanopoulou A S Moshé S L [Malnutrition and epilepsy] J Pediatr (Rio J) 2002 78 01 7 8 14647805

[5] Ramos A MF Nutritional impact of the ketogenic diet in difficult-to-control childhood refractory epilepsy (dissertation) São Paulo (SP): Universidade Federal de São Paulo; 2004

[6] Su S W Cilio M R Sogawa Y Silveira D C Holmes G L Stafstrom C E Timing of ketogenic diet initiation in an experimental epilepsy model Brain Res Dev Brain Res 2000 125(1-2):131 138 11154768 10.1016/s0165-3806(00)00130-9

[7] Sampaio L PB ABC of the ketogenic diet for refractory epilepsy. 1st edition Rio de Janeiro (RJ): DOC Content, 2018

[8] Hsieh D T Pfeifer H H Thiele E A Dietary management of epilepsy Am Fam Physician 2012 86 11 1000 1001 23198664

[9] Fernandes P Souza E Investigation protocols of psychological variables in infantile epilepsy Psicol, Teor Pesqui 2001 17 02 195 197

[10] Scheffer I E Berkovic S Capovilla G ILAE classification of the epilepsies: Position paper of the ILAE Commission for Classification and Terminology Epilepsia 2017 58 04 512 521 28276062 10.1111/epi.13709 PMC5386840

[11] Woodyatt R T Objects and method of diet adjustment in diabetics Arch Intern Med (Chic) 1921 28 125 141

[12] Wilder R M The effect on ketonemia on the course of epilepsy Mayo Clin Bull. 1921 2 307 308

[13] World Health Organization.Growth reference data for 5–19 years [Internet]Genebra: WHO,2007[cited 2021 Nov 15]. Available from:https://www.who.int/tools/growth-reference-data-for-5to19-years/indicators

[14] World Health Organization.Growth reference data for 0–5 years [Internet]Genebra: WHO,2006[cited 2021 Nov 15]. Available from:https://www.who.int/publications-detail-redirect/924154693X

[15] Summary of the second report of the National Cholesterol Education Program (NCEP) Expert Panel on Detection, Evaluation, and Treatment of High Blood Cholesterol in Adults (Adult Treatment Panel II) JAMA 1993 269 23 3015 3023 8501844

[16] Kwiterovich P Beyond Cholesterol. The John Hopkins Complete Guide Avoiding Heart Disease. 1st ed Maryland (BA): The John Hopkins Press; 1989 287 305

[17] Huttenlocher P R Ketonemia and seizures: metabolic and anticonvulsant effects of two ketogenic diets in childhood epilepsy Pediatr Res 1976 10 05 536 540 934725 10.1203/00006450-197605000-00006

[18] Freeman J M Kossoff E H Hartman A L The ketogenic diet: one decade later Pediatrics 2007 119 03 535 543 17332207 10.1542/peds.2006-2447

[19] de Kinderen R J Lambrechts D A Postulart D Research into the (Cost-) effectiveness of the ketogenic diet among children and adolescents with intractable epilepsy: design of a randomized controlled trial BMC Neurol 2011 11 10 21262002 10.1186/1471-2377-11-10 PMC3039580

[20] Schlindwein-Zanini R Portuguez M W Costa D Marroni S Costa J D Refractory epilepsy: rebound of quality of life of child and his caretaker J Epilepsy Clin Neurophysiol 2007 13 04 1980 5365

[21] Betting L E Kobayashi E Montenegro M A [Treatment of epilepsy: consensus of the Brazilian specialists] Arq Neuropsiquiatr 2003 61 04 1045 1070 14762617 10.1590/s0004-282x2003000600032

[22] Amorim P Padilha P Accioly E Ketogenic diet and applications in children with epilepsy care Rev Bras Nutr Clin 2013 28 01 45 53

[23] Thomas S V Bindu V B Psychosocial and economic problems of parents of children with epilepsy Seizure 1999 8 01 66 69 10091852 10.1053/seiz.1998.0241

[24] Martin-McGill K J Jackson C F Bresnahan R Levy R G Cooper P N Ketogenic diets for drug-resistant epilepsy Cochrane Database Syst Rev 2018 11 11 CD001903 30403286 10.1002/14651858.CD001903.pub4 PMC6517043

[25] Wijnen B FM de Kinderen R JA Lambrechts D AJE Long-term clinical outcomes and economic evaluation of the ketogenic diet versus care as usual in children and adolescents with intractable epilepsy Epilepsy Res 2017 132 91 99 28364726 10.1016/j.eplepsyres.2017.03.002

[26] Paoli A Ketogenic diet for obesity: friend or foe? Int J Environ Res Public Health 2014 11 02 2092 2107 24557522 10.3390/ijerph110202092 PMC3945587

[27] Vining E P Clinical efficacy of the ketogenic diet Epilepsy Res 1999 37 03 181 190 10584968 10.1016/s0920-1211(99)00070-4

[28] Klepper J Leiendecker B Heussinger N Lausch E Bosch F Severe Hypertriglyceridemia in Glut1D on Ketogenic Diet Neuropediatrics 2016 47 02 132 136 26902182 10.1055/s-0036-1572413

[29] Tagliabue A Bertoli S Trentani C Borrelli P Veggiotti P Effects of the ketogenic diet on nutritional status, resting energy expenditure, and substrate oxidation in patients with medically refractory epilepsy: a 6-month prospective observational study Clin Nutr 2012 31 02 246 249 22019282 10.1016/j.clnu.2011.09.012

[30] Vining E P Pyzik P McGrogan J Growth of children on the ketogenic diet Dev Med Child Neurol 2002 44 12 796 802 12455855 10.1017/s0012162201002961

[31] Groleau V Schall J I Stallings V A Bergqvist C A Long-term impact of the ketogenic diet on growth and resting energy expenditure in children with intractable epilepsy Dev Med Child Neurol 2014 56 09 898 904 24749520 10.1111/dmcn.12462 PMC4133288

[32] Mahan L K Arlin M T Food, Nutrition and Diet Therapy: Nutritional care in diseases of the Nervous System and Mental Disorders. 8th ed São Paulo (SP): Roca;1995 653 654

[33] Lehninger A L Nelson D L Cox M M Principles of Biochemistry. 5th ed Porto Alegre (RS):Artmed;2011 648 667

[34] Bergqvist A G Long-term monitoring of the ketogenic diet: Do's and Don'ts Epilepsy Res 2012 100 03 261 266 21855296 10.1016/j.eplepsyres.2011.05.020

[35] Schmidt D Baumgartner C Löscher W Seizure recurrence after planned discontinuation of antiepileptic drugs in seizure-free patients after epilepsy surgery: a review of current clinical experience Epilepsia 2004 45 02 179 186 14738426 10.1111/j.0013-9580.2004.37803.x

[36] Ramos A MF Ketogenic diet efficacy used in the treatment of children and adolescents refractory epilepsy Rev Neurociências 2001 9 03 127 131

[37] Freitas A Paz J A Casella E B Marques-Dias M J Ketogenic diet for the treatment of refractory epilepsy: a 10 year experience in children Arq Neuropsiquiatr 2007 65(2B, 2b)381 384 17665000 10.1590/s0004-282x2007000300003

[38] Freeman J M Kelly M T Freeman J B The epilepsy diet treatment. 1st ed New York (NY): Demos Medical Publishing; 1994

[39] Mackay M T Bicknell-Royle J Nation J Humphrey M Harvey A S The ketogenic diet in refractory childhood epilepsy J Paediatr Child Health 2005 41 07 353 357 16014140 10.1111/j.1440-1754.2005.00630.x

